# Fish consumption, fish oil supplements and risk of atherosclerosis in the Tromsø study

**DOI:** 10.1186/s12937-018-0364-8

**Published:** 2018-05-25

**Authors:** Stein Harald Johnsen, Bjarne K. Jacobsen, Sigrid K. Brækkan, John-Bjarne Hansen, Ellisiv B. Mathiesen

**Affiliations:** 1 Department of Neurology, University Hospital of North Norway, Sykehusvegen 38, 9019 Tromsø, Norway; 2 Brain and Circulation Research Group, Department of Clinical Medicine, UiT The Arctic University of Norway, Tromsø, Norway; 3 Department of Community Medicine, UiT The Arctic University of Norway, Tromsø, Norway; 4 K. G. Jebsen TREC - Thrombosis Research and Expertise Center, Department of Clinical Medicine, UiT The Arctic University of Norway, Tromsø, Norway; 5 Division of Internal Medicine, University Hospital of North Norway, Tromsø, Norway

**Keywords:** Atherosclerosis, Fish consumption, PUFAs, Carotid ultrasonography, Fish oil

## Abstract

**Background:**

Whether long-chain n–3 PUFAs of marine origin have an anti-atherogenic effect in the general population has hardly been studied. In this population-based study, we hypothesized that fatty fish and fish oil intake protect against development of novel atherosclerotic plaques and is associated with reduced plaque size.

**Methods:**

We obtained questionnaire-based information on fish consumption and carotid ultrasonography from 3900 persons aged 45–74 years. The questionnaires were validated by measuring serum concentrations of PUFAs and triglycerides in a subgroup. At follow-up seven years later, 2983 (76%) went through a second ultrasound scanning. Logistic regression and general linear models were used to analyze the outcome (plaque presence and plaque area) as a function of fish consumption, including analyses stratified on fish oil supplements.

**Results:**

At baseline, lean fish intake < 1 time/week vs. 1–1.9 times/week was associated with risk of plaque (OR 1.34, 95% CI 1.03–1.76). Fatty fish intake and use of fish oil supplements were not statistically significantly associated with atherosclerosis at baseline. In persons without plaque at baseline, total fish consumption ≥3 times/week vs. 1–1.9 times/week was associated with risk of novel plaque (OR 1.32, 95% CI 1.01–1.73) and larger plaque area (1.76 mm^2^ vs. 1.46 mm^2^, *p* = 0.02) at follow-up. Adjustments for use of fish oil supplements had no impact on the associations, and no interactions were seen between total, fatty or lean fish consumption and fish oil intake.

**Conclusions:**

We found no protective effect of fatty fish eating or fish oil supplements on atherosclerotic plaque formation or plaque area in a general population. Lean fish consumption was associated with a reduced risk for plaque in cross-sectional analysis, suggesting that the beneficial effects of fish consumption on atherosclerosis may be mediated through other mechanisms than n-3 PUFAs.

## Background

Epidemiological, animal, and cell culture studies show that long-chain n–3 PUFAs of marine origin, eicosapentaenoic acid (EPA), docosahexaenoic acid (DHA) and docosapentaenoic acid (DPA), reduce the risk of cardiovascular diseases (CVD). Fatty fish and fish oils are rich sources of these fatty acids. The most compelling evidence for cardiovascular benefits of n-3 PUFAs comes from 4 controlled trials of nearly 40,000 participants randomized to receive EPA with or without DHA in primary prevention [1], after myocardial infarction (MI) [2, 3], and in heart failure [4]. However, recent meta-analyses including newer randomized trials found that, except for a 10% reduction in sudden cardiac death among patients with prior MI, n-3 PUFA supplementation was not associated with a lower risk of all-cause mortality, non-fatal MI, or stroke [5, 6]. Taken together, the cumulative evidence suggests that n-3 PUFA supplements may reduce cardiac death in patients with prior MI, possibly through a reduction in ischemia-induced ventricular fibrillation [6]. In the recent updated advisory report, the American Heart Association has endorsed the use of n-3 PUFA at a dose of approximately 1 g/day, either in the form of fatty fish or fish oil supplements in patients with a previous MI or in patients with heart failure without preserved left ventricular function [6].

N − 3 PUFAs can influence many aspects of the pathogenesis of CVD, including inflammation [7], arrhythmias [8–12], platelet aggregation [13, 14], hypertension [15–17], and hyperlipidemia [18–20]. Although most evidence points to an antiarrhythmic effect, several studies have suggested that n-3 PUFAs may also have favorable effects on the pathogenesis of atherosclerosis. Fish oil has prevented the development of experimental atherosclerosis in pigs and rhesus monkeys [21–23]. In humans, a case-control study on peripheral arterial disease found that plasma levels of DPA related inversely to blocked arteries [24]. Cross-sectional studies with carotid ultrasonography revealed that carotid artery wall thickness decreased with EPA, DHA and DPA intake [25, 26]. A population based prospective study of atherosclerosis in men aged 40–49 years showed that serum concentrations of n-3 PUFAs contributed to the difference incidence of coronary artery calcification between Japanese and US white men [27].

Whether any anti-atherosclerotic effects of fish consumption is due to biological effects of n-3 PUFAs, or fish intake itself, due to replacement of other less favorable foods, has not been clearly addressed. An observational study in postmenopausal women found that after 3.2 years of follow-up, the consumption of ≥2 servings of fish or ≥ 1 serving of tuna or dark fish each week was associated with less progression of coronary artery stenosis [28]. A recent Italian study of 961 persons aged 18–89 y without clinically known atherosclerotic disease found that consuming ≥2 vs. < 1 serving of fish weekly was protective against carotid atherosclerosis defined as common carotid intima media thickness (cIMT) ≥ 0.9 mm or a focal plaque of > 1.2 mm [29]. According to The European Society of Cardiology (ESC)/European Society of Hypertension (ESH) guidelines (2013) SCORE Chart, asymptomatic vascular damage is defined as the presence of IMT > 0.9 mm or a focal plaque.

In this observational population-based study, we investigated the association between habitual fish consumption, fish oil consumption and carotid atherosclerosis defined as the presence of plaques on ultrasound assessment. We hypothesized that fatty fish and fish oil intake protect against development of novel atherosclerotic plaques and is associated with reduced plaque size (area) at baseline and follow-up.

## Methods

### Subjects

For this study, we used cross-sectional and prospective data from the 4th (1994–95) and 5th survey (2001) of the Tromsø study [30]. In the 4th survey (baseline), valid information on carotid plaque number, plaque area and fish consumption was obtained in 4589 persons aged 45–74. We excluded 689 persons with previous angina pectoris, MI or stroke, leaving 3900 eligible for the present study. Valid information on fish oil supplements was available in 3290 participants. In the 5th survey (follow-up), 2983 out of the 3900 subjects (76%) underwent a new valid ultrasound scanning, in whom 2535 persons had valid information on fish oil supplements.

### Ethics

The Norwegian Data Inspectorate licensed all data. The Regional Committee for Research Ethics approved the study (REC no. 200005785–7/IAY/400). All the participants gave their written informed consent.

### Assessment of fish consumption and fish oil supplements

At baseline, information of weekly consumption of fish for dinner and intake of fish oil supplements was assessed through a self-administered questionnaire. For participants aged < 70 years, the questionnaire covered 37 food frequency items, whereas those aged ≥70 years filled in a simplified questionnaire that covered only 23 food frequency items. Participants were asked how many times per week they usually ate fatty and lean fish, respectively (in the analyses categorized into four groups: never, < 1 time/week, 1 time/week, ≥2 times/week). Total weekly consumption of fish (times per week) was calculated by combining the answers given to the fatty and lean fish questions, and categorized into the following categories: < 1 time/week, 1–1.9 times/week, 2–2.9 times/week, and ≥ 3 times/week. According to the Norwegian Directorate of Health, a normal serving size of fish is 150 g. Participants were additionally asked to report how many months during the last year they had taken fish oil supplements on a daily basis. Intake of fish oil supplements was categorized as “no supplements” (no months during last year) and “supplements” (any number of months during the last year). The validity and reproducibility of dietary data from self-administered questionnaires in the Tromsø cohort have been reported previously [31, 32]. Additionally for this study, we validated the questionnaire based dietary assessment on fish servings and fish oil supplementation in a subgroup of 1656 participants by measuring serum concentrations of n-3 PUFAs and exploring the expected triglyceride-lowering effect of n-3 PUFAs [33].

### Cardiovascular risk factors

Height and weight were measured standardized. Body mass index (BMI) was calculated as weight in kilograms, divided by the square of height in meters (kg/m^2^). Specially trained personnel recorded blood pressure with an automatic device (Dinamap Vital Signs Monitor, Tampa Fla) with the participants in sitting position after 2 min rest. Three readings of blood pressure (on the upper right arm) were taken, separated with a 1-min interval. We used the average of the last two measurements in the analyses. Non-fasting serum total cholesterol and triglycerides were analyzed by enzymatic colorimetric methods with commercial kits (CHOD-PAP for cholesterol and GPO-PAP for triglycerides; Boehringer-Mannheim). Serum HDL cholesterol was measured after precipitation of lower-density lipoprotein with heparin and manganese chloride. LDL cholesterol was estimated using the Friedewald equation. Fibrinogen was measured with the PT-Fibrinogen reagent (Instrumentation Laboratory). Monocytes and white blood cells were counted with automated cell counters by standard techniques. CRP was analyzed in thawed aliquots after storage at − 70 °C with a particle-enhanced immunoturbidimetric assay on a Modular P with reagents from Roche Diagnostics (Roche, Mannheim, Germany). The Department of Clinical Chemistry, University Hospital of North Norway, did all the blood sample analyses. Information concerning education (in the analyses dichotomized to education at college/university level (yes/no)), leisure physical activity (sweating/out of breath ≥1 h/week (yes/no)), coffee consumption (cups/day), previous MI, prevalent angina pectoris and diabetes mellitus (yes/no), and cigarette smoking (never/previously/currently) was obtained from a questionnaire enclosed in the letter of invitation. We defined present coronary heart disease (CHD) as self-reported prevalent angina pectoris or previous MI.

### Ultrasonography and measures of atherosclerosis

The ultrasound methods and reproducibility of carotid examination have been detailed elsewhere [34, 35]. At baseline and follow-up, we used identical ultrasound imaging system (Acuson Xp10 128, ART upgraded, with a 7.5-MHz linear-array transducer, aperture size 38 mm). Six locations in the right carotid artery (the far and near walls of the common carotid, the bulb, and the internal carotid) were scanned for the presence of plaque (yes/no) and the number of plaques were registered. In Adobe Photoshop v7.0, we analyzed still images of each plaque, and the area (mm^2^) was calculated. In participants with more than one plaque, the sum of plaque areas was taken as the total plaque area (TPA). Novel plaque formation was defined as ≥ one new plaque at follow-up in persons with no plaque at baseline. The difference in TPA between baseline and follow-up (ΔTPA) was calculated.

### Statistics

Descriptive statistics (means or percentages) were computed for each independent baseline variable in categories of fish consumption. Presence of plaque and TPA were the dependent variables in the regression models. TPA at baseline and follow-up (but not ΔTPA) was square-root-transformed to approximate normal distribution. Fish consumption (total, fatty fish and lean fish) was the main explanatory variable. Predefined risk factors for atherosclerosis (systolic blood pressure, current smoking, total cholesterol, HDL-cholesterol, triglycerides, diabetes, leucocyte count, monocyte count, fibrinogen, CRP and anti-hypertensive medication) were introduced as co-variables to adjust for confounding. First, we analyzed the whole sample on fish consumption without fish oil in the models. Analyses were then repeated in models stratified by fish oil supplements (yes/no). Logistic regression and general linear models were used to model the outcome (plaque presence and TPA, respectively) as a function of fish consumption. Linear trends across level of fish intake were tested by logistic regression for categorical dependent variables and by linear regression for continuous dependent variables. Statistical analyses were conducted with SAS version 9.4 software (SAS Institute, Cary, NC), and two-sided *P* <  0.05 was considered statistically significant.

## Results

Table 1 lists baseline characteristics in the different categories of fish servings. Elderly men consumed more fish than relatively younger women did. There was an inverse trend between the number of fish servings per week and serum triglycerides (*p* = 0.002). Leucocyte count was positively associated with fish consumption (*p* = 0.05). Participants who consumed fatty fish ≥2 times/week (*n* = 608) were older, more likely to be males, daily smokers, used more fish oil supplements and lipid-lowering drugs, and had higher levels of CRP (Table 2) than subjects with less frequent fatty fish intake. The use of fish oil supplements was associated with female sex, higher education, physical activity, not being a daily smoker, less boiled coffee drinking, as well as lower BMI, serum triglycerides, monocyte concentration, white cell count, fibrinogen and CRP (Table 3).

**Table 1:** Distribution of baseline characteristics across categories of fish consumption: the Tromsø Study

	Fish servings, times per week				
Baseline characteristics	< 1 *n* = 258	1–1.9 *n* = 1325	2–2.9 *n* = 977	≥ 3 *n* = 1340	p-trend
Age, y	58.4 (0.4)	59.3 (0.2)	61.8 (0.2)	63.0 (0.2)	< 0.0001
Male, %	40.8	46.6	54.1	55.0	< 0.0001
Higher education, %^a^	16.9	20.3	17.6	18.9	0.8
Physical active, %	19.8	23.0	24.5	23.4	0.4
BMI, kg/m^2^	26.4 (0.2)	25.9 (0.1)	25.9 (0.1)	26.1 (0.1)	0.9
Total cholesterol, mmol/L	6.83 (0.08)	6.77 (0.03)	6.79 (0.04)	6.78 (0.03)	0.9
LDL cholesterol, mmol/L	4.48 (0.07)	4.44 (0.03)	4.46 (0.04)	4.48 (0.03)	0.7
HDL cholesterol, mmol/L	1.50 (0.03)	1.56 (0.01)	1.57 (0.01)	1.57 (0.01)	0.07
LDL/HDL ratio	3.20 (0.07)	3.12 (0.03)	3.05 (0.04)	3.10 (0.03)	0.3
Triglycerides, mmol/L	1.85 (0.06)	1.68 (0.03)	1.66 (0.03)	1.61 (0.03)	0.002
Systolic blood pressure, mmHg	146.7 (1.3)	144.4 (0.6)	146.2 (0.7)	145.5 (0.6)	0.6
Diastolic blood pressure, mmHg	85.2 (0.8)	83.4 (0.3)	84.4 (0.4)	83.9 (0.3)	0.9
Hypertension, %	60.2	53.9	56.9	56.4	0.7
Current smoking, %	29.3	31.1	31.2	33.7	0.09
Diabetes, %	2.5	2.8	1.7	2.8	0.9
Fish oil supplements, %^b^	45.6	50.5	45.1	52.4	0.2
Antihypertensive medication, %	11.7	10.3	10.7	9.8	0.4
Lipid-lowering medication, %^c^	0.8	1.7	1.1	1.9	0.4
Leukocyte count, × 10^9^/L	6.76 (0.12)	6.84 (0.05)	6.95 (0.06)	6.95 (0.05)	0.048
Monocyte count, × 10^9^/L	0.59 (0.01)	0.58 (0.01)	0.59 (0.01)	0.59 (0.01)	0.2
Fibrinogen, g/L	3.39 (0.05)	3.38 (0.02)	3.37 (0.03)	3.40 (0.02)	0.7
LogCRP, mg/ml	0.31 (0.07)	0.26 (0.03)	0.22 (0.04)	0.26 (0.03)	0.6

Age was sex-adjusted and sex was age-adjusted. All other variables were age- and sex-adjusted means (SE) or %

*BMI* body mass index

^a^College or university

^b^610 (15.6%) participants had missing information on fish oil supplements

^c^Participants < 70 years

**Table 2:** Baseline characteristics stratified by fatty fish consumption: the Tromsø study

	Fatty fish servings, times per week			
Baseline characteristics	< 1 *n* = 1841	1–1.9 *n* = 1518	≥2 *n* = 541	p-trend
Age, y	60.3 (0.2)	61.3 (0.2)	63.6 (0.3)	< 0.0001
Male, %	49.6	50.4	57.1	0.009
Higher education, %^a^	18.2	19.9	18.3	0.6
Physical active, %	23.2	23.0	24.4	0.7
BMI, kg/m^2^	25.9 (0.1)	26.0 (0.1)	26.1 (0.2)	0.4
Total cholesterol, mmol/L	6.77 (0.03)	6.80 (0.03)	6.77 (0.05)	0.9
LDL cholesterol, mmol/L	4.45 (0.03)	4.48 (0.03)	4.46 (0.05)	0.8
HDL cholesterol, mmol/L	1.55 (0.09)	1.57 (0.01)	1.56 (0.02)	0.3
LDL/HDL ratio	3.10 (0.03)	3.09 (0.03)	3.13 (0.05)	0.9
Triglycerides, mmol/L	1.69 (0.02)	1.65 (0.03)	1.63 (0.04)	0.2
Systolic blood pressure, mmHg	145.4 (0.5)	145.1 (0.5)	146.1 (0.9)	0.7
Diastolic blood pressure, mmHg	84.0 (0.3)	83.6 (0.3)	84.6 (0.5)	0.7
Hypertension, %	55.8	55.3	58.3	0.5
Current smoking, %	30.7	32.0	35.7	0.04
Coffee consumption, cups/d				
Boiled coffee	3.1 (0.1)	2.9 (0.1)	3.1 (0.2)	0.5
Other coffee	2.7 (0.1)	2.7 (0.1)	2.7 (0.1)	0.8
Diabetes, %	2.1	2.9	2.9	0.2
Fish oil supplements, %^b^	45.8	52.0	55.1	< 0.0001
Antihypertensive medication, %	11.0	9.1	11.5	0.7
Lipid-lowering medication, %^c^	1.1	1.9	2.7	0.02
Leucocyte count, × 10^9^/L	6.86 (0.04)	6.89 (0.05)	7.05 (0.08)	0.08
Monocyte count, × 10^9^/L	0.58 (0.004)	0.59 (0.005)	0.60 (0.008)	0.3
Fibrinogen, g/L	3.37 (0.02)	3.38 (0.02)	3.44 (0.04)	0.1
logCRP, mg/L	0.23 (0.03)	0.26 (0.03)	0.35 (0.05)	0.04

Age was sex-adjusted and sex was age- adjusted. All other variables were age- and sex-adjusted means (SE) or %

*BMI* body mass index

^a^College or university

^b^610 (15.6%) participants had missing information on fish oil supplements

^c^Participants < 70 years

**Table 3:** Baseline characteristics in users and non-users of fish oil supplements: the Tromsø Study

	Fish oil supplements		
	Yes *n* = 1627	No *n* = 1663	p
Age, y	61.4 (0.2)	60.6 (0.2)	0.0007
Male, %	49.0	55.7	0.0001
Higher education, %^a^	25.0	16.0	< 0.0001
Physical active, %	27.2	23.0	0.005
BMI, kg/m^2^	25.6 (0.1)	26.3 (0.1)	< 0.0001
Total cholesterol, mmol/L	6.73 (0.03)	6.80 (0.03)	0.09
LDL cholesterol, mmol/L	4.43 (0.03)	4.48 (0.03)	0.2
HDL cholesterol, mmol/L	1.57 (0.01)	1.55 (0.01)	0.3
LDL/HDL ratio	3.09 (0.03)	3.12 (0.09)	0.4
Triglycerides, mmol/L	1.61 (0.03)	1.70 (0.03)	0.02
Systolic blood pressure, mmHg	144.6 (0.5)	145.4 (0.5)	0.2
Diastolic blood pressure, mmHg	83.5 (0.3)	84.0 (0.3)	0.2
Hypertension, %	54.1	55.8	0.3
Current smoking, %	28.7	34.8	0.0002
Coffee consumption, cups/d			
Boiled coffee	2.3 (0.09)	3.5 (0.09)	< 0.0001
Other coffee	2.9 (0.08)	2.6 (0.08)	0.009
Diabetes, %	2.1	2.4	0.6
Antihypertensive medication, %	10.4	9.5	0.4
Lipid-lowering medication, %^b^	2.0	1.1	0.04
Leucocyte count, × 10^9^/L	6.80 (0.05)	6.95 (0.05)	0.02
Monocyte count, × 10^9^/L	0.58 (0.004)	0.59 (0.004)	0.04
Fibrinogen, g/L	3.33 (0.02)	3.40 (0.02)	0.01
LogCRP, mg/L	0.20 (0.03)	0.27 (0.03)	0.04

Age was sex-adjusted and sex was age- adjusted. All other variables were age- and sex-adjusted means (SE) or %

*BMI* body mass index

^a^College or university

^b^Participants < 70 years

### Fish consumption and risk for plaque and TPA at baseline

At baseline, at least one plaque was present in 1884 of the 3900 (48.3%) persons. There was a statistically significant inverse trend (*p* = 0.02) between lean fish intake and plaque prevalence (but not TPA). Lean fish intake < 1 time/week vs. 1–1.9 times/week was associated with 34% increased risk of plaque (OR 1.34, 95% CI 1.03–1.76), whereas no significant associations were found between total or fatty fish intake and plaque prevalence or TPA (Table 4). Among the 3290 persons with available information about the use of fish oil supplements, the age- and sex-adjusted plaque prevalence (47.3% vs. 47.9%, *p* = 0.7) and square-root-transformed TPA levels (1.92 vs. 2.01 mm^2,^ *p* = 0.3) were similar in users and non-users.

**Table 4:** Risk for plaque and total plaque area at baseline according to categories of fish consumption: the Tromsø Study

Fish servings (times/wk)	N 3900	Plaque N (%)	Risk for plaque OR (95% CI)	TPA^a^ mm^2^ (SE)
Total fish				
< 1	258	124 (48.1)	1.29 (0.95–1.74)	2.17 (0.14)
1–1.9	1325	594 (44.8)	1 (Reference)	2.01 (0.06)
2–2.9	977	459 (47.0)	0.86 (0.71–1.04)	1.86 (0.07)
≥ 3	1340	707 (52.8)	1.01 (0.84–1.20)	2.03 (0.06)
p-trend			0.4	0.7
Fatty fish				
< 1	1841	832 (45.2)	0.95 (0.82–1.11)	1.94 (0.05)
1–1.9	1518	738 (48.6)	1 (Reference)	1.99 (0.06)
≥ 2	541	314 (58.0)	1.20 (0.96–1.51)	2.16 (0.10)
p-trend			0.06	0.07
Lean fish				
< 1	330	163 (49.4)	1.34 (1.03–1.76)	2.18 (0.13)
1–1.9	1437	652 (45.4)	1 (Reference)	1.99 (0.06)
≥ 2	2133	1069 (50.1)	0.93 (0.80–1.09)	1.96 (0.05)
p –trend			0. 02	0.2

Models were adjusted for age, sex, systolic blood pressure, current smoking, total cholesterol, HDL-cholesterol, triglycerides, diabetes, leucocyte count, monocyte count, fibrinogen, CRP and anti-hypertensive medication

*TPA* Total plaque area

^a^Square root transformed

### Fish consumption and plaque at follow-up

At follow-up, at least one plaque was present in 1869 of the 2983 (62.7%) persons who had a new ultrasound scan. This includes subjects with a plaque present already at baseline and subjects with a novel plaque. Both total fish (p-trend = 0.05) and fatty fish (p-trend = 0.02) consumption were positively associated with plaque prevalence (but not with TPA). No associations were found between lean fish consumption and plaque prevalence or TPA (results not shown in table). Among the 2535 persons with available information on fish oil supplements, the plaque prevalence (62.0% vs. 62.4%, *p* = 0.8) and square-root-transformed TPA levels (2.73 vs. 2.84 mm^2^, *p* = 0.3) were similar in users and non-users. Also, the change in TPA (Δ TPA) between baseline and follow-up (5.84 vs. 6.00 mm^2^, p = 0.8) was similar in users and non-users of fish oil supplements.

Of the 2016 persons with no plaque at baseline, 1634 (81%) were re-examined at follow-up, At least one novel plaque had developed in 700 (42.8%) of these. The risk of novel plaque and TPA increased linearly by number of total fish servings (Table 5). Total fish intake ≥3 times/week vs. 1–1.9 times/week was associated with 32% higher risk of novel plaque (OR 1.32, 95% CI 1.01–1.73) and larger TPA (1.76 mm^2^ vs. 1.46 mm^2^). Among the 1400 persons with available information on use of fish oil supplements, the prevalence of novel plaque (44.0% vs. 41.7%, *p* = 0.4) and square-root-transformed TPA level (1.63 vs. 1.62 mm^2^, *p* = 0.9) were similar in users and non-users of fish oil supplements.

**Table 5:** Risk for plaque and total plaque area at follow-up in persons with no plaque present at baseline, according to categories of fish consumption: the Tromsø Study

Fish servings (times/wk)	N 1634	Novel plaque N (%)	Risk for plaque OR (95% CI)	TPA^a^ mm^2^ (SE)
Total fish				
< 1	116	44 (37.9)	0.98 (0.62–1.54)	1.52 (0.19)
1–1.9	596	225 (37.8)	1 (Reference)	1.46 (0.08)
2–2.9	416	181 (43.5)	1.15 (0.87–1.52)	1.62 (0.10)
≥ 3	506	250 (49.4)	1.32 (1.01–1.73)	1.76 (0.09)
p-trend			0.04	0.02
Fatty fish				
< 1	829	332 (40.1)	0.90 (0.72–1.13)	1.54 (0.07)
1–1.9	631	280 (44.4)	1 (Reference)	1.63 (0.08)
≥ 2	174	88 (50.6)	1.18 (0.81–1.72)	1.75 (0.16)
p-trend			0.1	0.2
Lean fish				
< 1	142	55 (38.7)	0.99 (0.65–1.49)	1.57 (0.17)
1–1.9	636	253 (39.8)	1 (Reference)	1.52 (0.08)
≥ 2	856	392 (45.8)	1.12 (0.89–1.41)	1.66 (0.07)
p –trend			0.4	0.3

Models were adjusted for age, sex, systolic blood pressure, current smoking, total cholesterol, HDL-cholesterol, triglycerides, diabetes, leucocyte count, monocyte count, fibrinogen, CRP and anti-hypertensive medication

*TPA* Total plaque area

^a^Square root transformed

### Analyses adjusted for fish oil supplements

All analyses above were repeated in models restricted to subjects with available information about the use of fish oil supplements. Adjustments for this variable had no impact on the associations, and no interactions were seen between total, fatty or lean fish consumption and fish oil intake.

## Discussion

In this study, we found no protective effect of fatty fish intake or fish oil supplements on atherosclerosis assessed by carotid ultrasound. If anything, the association was positive. At baseline (Table 4), we found that lean fish consumption, if anything, reduced the odds of having carotid plaque. However, in prospective analyses, this finding could not be reproduced and total fish intake was actually associated with an increased risk of novel plaque formation in persons without pre-existing plaque.

As expected, we found an inverse trend between total fish consumption and serum triglycerides (Table 1) and that fish oil supplements were associated with low triglycerides (Table 3). However, no statistically significant inverse relationship was found between the intake of fatty fish and serum triglycerides (Table 2). It is well known that smoking increases serum triglyceride levels. In our study, the age- and sex adjusted triglyceride levels in smokers and non-smokers were 1.71 and 1.64 mmol/L, respectively (*p* = 0.06). Thus, the higher proportion of smokers in the high fatty fish consumption categories could potentially explain the lack of inverse association between fatty fish intake and triglyceride levels. High triglycerides are not directly atherogenic, but represent an important biomarker of cardiovascular risk because of their association with atherogenic VLDL remnant particles and apo C-III, a proinflammatory, proatherogenic protein found on all classes of the plasma lipoproteins [36].

Unexpectedly, we found no protective effect of fatty fish intake or fish oil supplements, both containing high amounts of n-3 PUFAs, on the risk for atherosclerosis. The evidence for an anti-atherogenic effect of n-3 PUFAs is admittedly not very strong. The studies that have shown effects are in general small, cross-sectional, have reported on cIMT and not plaque, or have been conducted in patients with previous CVD or high risk for CVD. A small randomized trial (*n* = 59) did not detect significant effects of fish oil supplementation (6 g/day for 2.3 years) on the progression of coronary atherosclerosis as measured by coronary angiography [37]. A larger randomized trial (*n* = 223) found modest effects of fish oil supplementation (1.65 g/day for two years) on coronary atherosclerosis as measured by coronary angiography [38], but not on carotid atherosclerosis as measured by ultrasound [39]. A third open-label randomized trial evaluated the effects of EPA (1.8 g/day for 2.1 years) on progression of carotid atherosclerosis in 81 Japanese patients with type 2 diabetes. Individuals randomized to EPA had less progression of both mean and maximal intimal medial thickness [40]. Although these small clinical studies suggest that fish oil consumption may modestly reduce progression of atherosclerosis, we could not reproduce this effect in a large general population. When looking closer into the 541 participants who consumed fatty fish ≥2 times/week (Table 2), we found that they were older, were more likely to be males and smokers, and had higher levels of CRP. On the other hand, users of fish oil supplement had a more favorable risk factor profile compared to non-users, implying that those who take fish oil supplements have a healthier lifestyle.

An interesting question is whether fish consumption itself, independent of n-3 PUFAs, can protect against atherosclerosis due to replacement of other less healthy foods. It is now increasingly recognized that the beneficial effects of fish consumption is not limited to lipids and fatty acids, but that the peptides and amino acids like taurine and glycine, together with vitamins, are also important for disease prevention [41]. Lean fish contains less energy than fatty fish and has been associated with less risk for metabolic syndrome [42]. At baseline, we found that consumption of lean fish, which contains very low levels of n-3 PUFAs, was inversely associated to plaque prevalence, and that the effect remained significant after adjustment for fish oil supplements. These findings suggest that mechanisms other than those mediated through fatty acids from fish consumption may be of importance.

Studies have suggested that the preparation method, frying in particular, may substantially alter the fatty acid content of a fishmeal [43]. A study by Mozaffarian et al. found that fried fish and fish sandwich consumption was not associated with lower risk for ischemic heart disease, arrhythmic death, or nonfatal myocardial infarction but rather with trends towards higher risk [44]. In addition, data from the Multi-Ethnic Study of Atherosclerosis showed that the dietary intake of non-fried fish was inversely associated with the prevalence of subclinical atherosclerosis defined as thickening of the common carotid intima media thickness [45]. Unfortunately, we lack specific registrations of differences in fish cooking procedures and nutrients that may influence the impact of fish intake.

Strengths of this study include the prospective design and a large general population with high attendance rate. As persons with cardiovascular diseases are more likely to have carotid plaque when compared to those without and at the same time might eat more fish for their vascular health, we excluded all participants with previous angina, MI and stroke to minimize confounding. We further validated the reliability of dietary assessment on fish servings and fish oil supplementation by measuring serum concentrations of n–3 PUFAs and exploring the expected triglyceride-lowering effect of n–3 PUFAs. Plaque presence and plaque area are considered as more robust measures of subclinical atherosclerosis than cIMT [46].

The study also has limitations, however. First, residual confounding can be expected. Even though we excluded persons with previous cardiovascular diseases, and made multiple adjustments, differences in lifestyle between groups may have biased the associations between fish consumption and atherosclerosis. Second, particularly regarding the relationships between fish intake and the risk of novel plaques, several of the risk factors may have changed over time, including fish consumption, which may lead to non-differential misclassification, and thereby underestimation of the true associations. Third, we lack specific registrations of differences in fish cooking procedures and nutrients that may influence the impact of fish intake. Even though we excluded persons older than 74 years, the use of a self-report questionnaire to obtain dietary intake data may be subject to recall bias, thus introducing error into our estimated models. A weakness of the study is related to loss of follow-up, which may have attenuated the results. Although we used a standardized protocol for the measurement of plaque at both baseline and follow-up, plaque assessment may be distorted by measurement error. Plaques of low echogenicity may have been overlooked. Any such misclassification is expected to underestimate the true association.

## Conclusions

In conclusion, we found no protective effect of fatty fish consumption or use of fish oil supplements on risk of atherosclerosis. In cross-sectional analyses, we found a preventive effect of lean fish eating on atherosclerotic plaque prevalence. The findings suggest that the beneficial effects of fish consumption on atherosclerosis may be mediated through other mechanisms than n-3 PUFAs.

## Abbreviations

BMI: Body mass index
CHD: Coronary heart disease
cIMT: Carotid intima media thickness
CRP: C-reactive protein
CVD: Cardiovascular diseases
DHA: Docosahexaenoic acid
DPA: Docosapentaenoic acid
EPA: Eicosapentaenoic acid
HDL: High density lipoprotein
LDL: Low density lipoprotein
MI: Myocardial infarction
PUFA: Polyunsaturated fatty acids
TPA: Total plaque area
VLDL: Very low density lipoprotein
